# Preoperative Vascular Function Assessment and Perioperative Vascular Events in Patients Posted for Elective Non-cardiac Surgeries

**DOI:** 10.7759/cureus.26188

**Published:** 2022-06-22

**Authors:** Ajeetviswanath Thanjavur Prabhakaran, Suman L Gupta, Prasanna U Bidkar, Ajith Ananthakrishnapillai, Srinivasan Swaminathan

**Affiliations:** 1 Anesthesiology and Critical Care, All India Institute of Medical Sciences, New Delhi, IND; 2 Anesthesiology, Jawaharlal Institute of Postgraduate Medical Education & Research, Puducherry, IND; 3 Anesthesiology and Critical Care, Jawaharlal Institute of Postgraduate Medical Education & Research, Puducherry, IND; 4 Cardiology, Jawaharlal Institute of Postgraduate Medical Education & Research, Puducherry, IND

**Keywords:** usg, mace, flow mediated vasodilation, carotid artery intima thickness, endothelial dysfunction

## Abstract

Background

Coronary atherosclerosis is usually asymptomatic until a major cardiac event occurs. Surgery is one of the major stress factors that play a role in hastening vascular deterioration in susceptible patients. Non-invasive tests to detect atherosclerosis and endothelial dysfunction have started gaining popularity nowadays, and of the several options, carotid artery intima-media thickness (IMT) and radial artery flow-mediated dilation (FMD) are two promising tests for detecting cardiovascular impairment.

Methods

This was a pilot study that was undertaken on 100 patients in a tertiary care medical center (Jawaharlal Institute of Postgraduate Medical Education & Research, Puducherry) between June 2015 and August 2016 with the aim of studying the prevalence of endothelial dysfunction and early atherosclerosis in the given population, and to find out the predictive power of preoperative vascular functional assessment in the prediction of perioperative cardiovascular events in the same population. We had selected patients who had at least two risk factors for endothelial dysfunction and were posted for elective non-cardiac surgical procedures via convenience sampling. Flow-mediated vasodilatation of the radial artery (FMD) and carotid intima-media thickness (CIMT) were measured on the previous day of surgery, while a fasting lipid profile was collected from the patients on the morning of the surgery. Endothelial dysfunction was defined as FMD<4.5%, while atherosclerosis was defined as CIMT>0.07 cm. Demographic details and baseline hemodynamic parameters of the patients were also noted preoperatively as well as intra-operatively, and patients were followed up for any major clinical adverse cardiovascular event post-operatively till they were discharged from the hospital.

Results

It was found that the prevalence of endothelial dysfunction was 23%, while the prevalence of early atherosclerosis was 33% in our study population. However, it was found that FMD and CIMT did not correlate with each other significantly, nor did they correlate significantly with perioperative cardiovascular events. The risk factors of the patients also did not correlate with the FMD and CIMT values of the patients in which they were impaired. Moreover, they did not have any significant correlation with the perioperative events that occurred.

Conclusion

The prevalence of endothelial dysfunction in our tertiary center was found to be 23%, and the prevalence of atherosclerosis was 33% in patients posted for elective non-cardiac surgery who had multiple risk factors. It was also found that non-invasive preoperative vascular assessment was not quite effective as hypothesized in predicting perioperative cardiovascular events.

## Introduction

Endothelial dysfunction, by itself, is a major causative factor for atherosclerosis [1]. It impairs vasomotor tone and promotes arterial thrombosis as well as causes migration and proliferation of vascular cells [2]. Thus, it has the potential to be used as a reversible and early marker of cardiovascular disease. Furthermore, the prediction of coronary artery disease prospectively can be made by the assessment of endothelial dysfunction even before arteries develop atherosclerotic changes.

Coronary atherosclerosis usually does not manifest with any symptoms until a major cardiac event occurs, which may even cause mortality. Surgery is one of the major stress factors that play a role in hastening vascular deterioration in susceptible patients. Non-invasive tests to detect atherosclerosis and endothelial dysfunction as early as possible have started gaining popularity nowadays as they are patient-friendly and easy to perform. Of the several options, carotid artery intima-media thickness (CIMT) and radial artery flow-mediated dilation (FMD) are two promising tests for detecting cardiovascular impairment. Impaired CIMT and FMD, which correlate with endothelial dysfunction, are also associated with subclinical hypothyroidism and rheumatoid arthritis [3,4]

The novelty of this study lay in the fact that it aimed to detect the effect of a short-term stress factor, such as surgery, on the cardiovascular system of the patients at risk using only CIMT and FMD impairment. Previous studies on a similar line have only studied long-term vascular events and their correlation with endothelial function.

## Materials and methods

**Figure 1:**
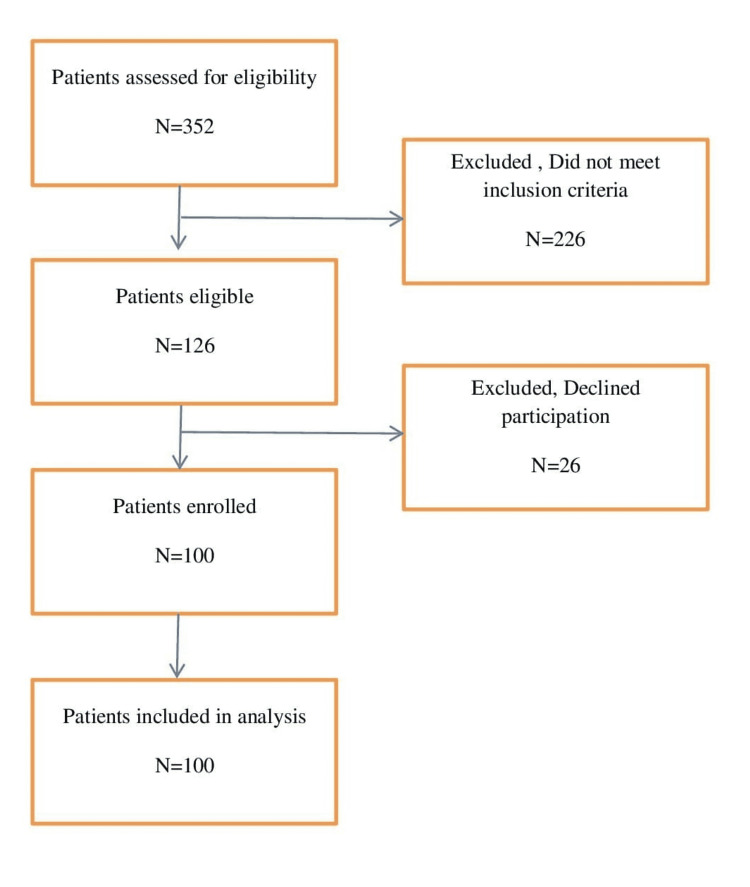
STROBE diagram STROBE - Strengthening the Reporting of Observational Studies in Epidemiology

This observational study was undertaken in the Department of Anesthesiology and Critical care, Jawaharlal Institute of Postgraduate Medical Education & Research (JIPMER), Puducherry, between June 2015 and August 2016. After the review and approval from the institute ethics committee and registration with the Clinical Trials Registry of India, enrollment of patients was done. This was a feasibility study with a convenience sampling technique with written consent being taken from 100 patients scheduled for elective non-cardiac surgeries. They belonged to the age group of 18-65 years, with at least two or more risk factors for endothelial dysfunction [5], including hypercholesterolemia, hypertension, diabetes mellitus, chronic smoking, and a history of ischemic heart disease. Pregnant patients, patients undergoing vascular surgery, and patients who were not willing to participate in the study were excluded (Figure 1).

Enrolled patients were explained about the study, and informed consent was obtained. Their pre-anesthetic checkup was done on the previous day of the surgery when their baseline hemodynamic parameters and BMI were noted. The FMD of the radial artery and the intima-media thickness (IMT) of the carotid artery were then measured using a 7-10 MHz linear ultrasound probe from Sonosite® (FUJIFILM Sonosite, Inc., Bothell, Washington) after allowing the patients to rest for 10 minutes to ensure stable conditions before scanning.

**Figure 2:**
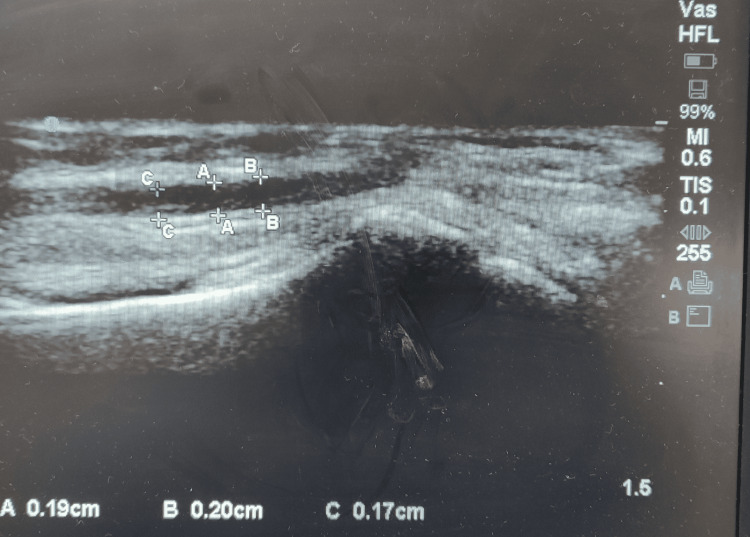
Measurement of radial artery flow-mediated vasodilatation A,B,C - three different readings are taken and an average value is taken

Longitudinal images of the radial artery were obtained from the patients in the supine position, with their arms resting by their side. Baseline radial artery diameter measurements were obtained in end-diastole (R-wave of the ECG) (Figure 2). The blood pressure (BP) cuff was placed proximally to the radial artery on the arm and then inflated to 100 mm Hg above systolic pressure for five minutes. The artery caliber was then measured using the same probe, and the values were recorded in terms of percentage increase or decrease in caliber. FMD was considered to be impaired [6] when the value was less than 4.5% [7].

**Figure 3:**
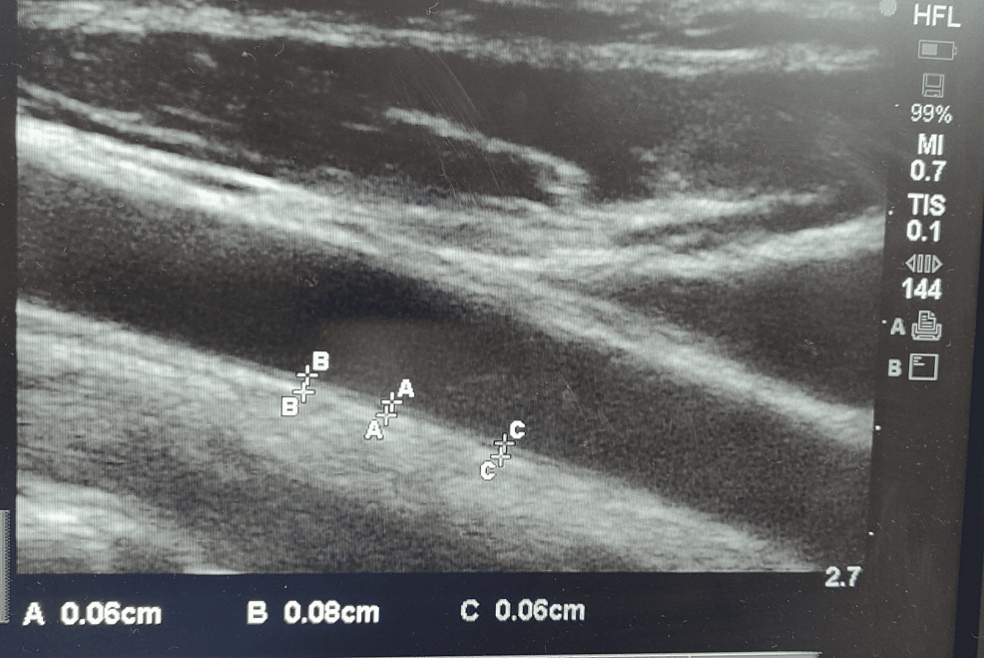
Measurement of carotid artery intima-media thickness A,B,C - a total of three readings are taken and an average value is taken

To measure carotid intima-media thickness, ultrasonography (USG) of the right carotid artery was performed with the same probe. On a longitudinal 2D USG image of the carotid artery, two bright white lines with a space in between were visualized, which were the anterior (intima) and posterior (media) walls of the carotid artery (Figure 3). The distance between the two white lines was recorded as the intima-media thickness. The subjects were in a sitting position when the measurements were made. The normal IMT value was taken as </=0.07 cm [8].

A fasting lipid profile was obtained from the patients on the morning of the surgery. Regional or GA or both, depending upon the type of surgery, were given under optimum conditions using standard procedures. The patients were then followed up intraoperatively using standard anesthetic monitoring and post-operatively till discharge for any clinical cardiac or vascular events based on the MACE criteria [9], which included: death, acute coronary syndrome, arrhythmias, or stroke.

The data was analyzed using statistical software SPSS version 19.0 (IBM Inc., Armonk, New York). The distribution of data on continuous variables was expressed as mean ± SD. These include age, BMI, carotid intima-media thickness (CIMT), and fasting lipid profile (FLP). The distribution of data on categorical variables was expressed as frequency (%). These include impaired FMD (<4.5%), impaired CIMT (>0.07cm), perioperative cardiac events, and type of surgery. FMD was expressed as median with IQR. The median FMD between two groups, defined by the presence or absence of clinical risk factors, was compared using the Mann-Whitney U test, while the mean CIMT between the same two groups was compared using the Student’s t-test. Association between impaired FMD and CIMT with the occurrence of perioperative cardiac events was tested using Fisher’s exact test. The correlation between FMD and CIMT was assessed using Pearson’s correlation. The odds ratio was calculated to study the association between the clinical risk factors and the occurrence of perioperative cardiac events and also to study the association between the risk factors and the presence of impaired FMD and CIMT.

All statistical analyses were carried out at a 5% level of significance, and p<0.05 was considered significant.

## Results

The mean age distribution of patients who took part in this study was 47.72±9.03 years. Sixty-two patients were male, while the remaining 38 were female. Out of the 100 patients chosen, 29 were overweight, and 13 were obese, with a mean BMI of 25.30±4.58 (Table 1). The prevalence of endothelial dysfunction was found to be 23%, and the prevalence of early atherosclerosis was found to be 33% in our study population. 11% of the population had both endothelial dysfunction and early atherosclerosis (Table 2).

**Table 1:** Demographics MAP - mean arterial pressure, LDL - low-density lipoprotein, HDL - high-density lipoprotein, VLDL - very low density lipoprotein

Patient demographics	
Age (mean ±SD)	47.72±9.03 years
Gender (male/demale)	62%/38%
BMI (mean ±SD)	25.30±4.58 kg/m2
Duration of surgery (mean ±SD)	2.0 ± 0.32 hours
Type of anesthesia (general/regional/combined)	45%/ 49%/ 6%
Risk of surgery (high/low)	16% / 84%
Pre-op MAP (mean ±SD)	105.36±6.52 mmHg
Pre-op heart rate (mean ±SD)	78.8±11.07 beats/min
Total cholesterol (mean ±SD)	177.35±28.01 mg/dl
LDL (mean ±SD)	98.11±22.94 mg/dl
HDL (mean ±SD)	45.03±5.87 mg/dl
VLDL (mean ±SD)	34.21±8.46 mg/dl
Triglycerides (mean ±SD)	171.05±42.30 mg/dl

**Table 2:** Prevalence of endothelial dysfunction FMD - flow-mediated dilation, CIMT - carotid intima-media thickness

Parameter	Value	No. of patients with impairment	Cardiovascular events observed (p-value)
Median FMD	7.36% (4.67-9.09)	23	3 (p=0.133)
Mean CIMT	0.067 ± 0.015 cm	33	3 (p=0.393)
Both impaired		11	1 (p=0.512)

Six patients had intraoperative cardiac events, while there were no major adverse cardiovascular events in the post-operative period. The occurrence of perioperative cardiac events was not significant in patients with impaired FMD, impaired CIMT, or in patients with both impaired. There was no significant association between any of the five clinical risk factors, namely diabetes mellitus, hypertension, ischemic heart disease, hypercholesterolemia, and smoking, with endothelial dysfunction or early atherosclerosis in our study population. The patients' baseline metabolic equivalents were greater than four so as to make them eligible for elective surgeries. The odds of the clinical risk factors leading to the cardiac events were statistically insignificant (Table 3). There was no statistically significant difference in the occurrence of intraoperative cardiac events among high-risk and low-risk surgeries in the study population.

**Table 3:** Odds ratio of each clinical risk factor leading to adverse cardiovascular events

Risk factor	No. of patients with impaired FMD (<4.5%)	No. of patients with early atherosclerosis (CIMT>.07cm)	No. of patients with cardiovascular events (p-value)
Diabetes mellitus	21 (p=0.954)	25 (p=0.181)	5 (p=0.982)
Hypertension	17 (p=0.725)	25 (p=0.463)	5 (p=0.501)
Ischemic heart disease	-	3 (p=0.830)	-
Hypercholesterolemia	-	1 (p=0.620)	1 (p=0.285)
Smoking history	9 (p=0.720)	13 (p=0.396)	2 (p=0.888)
High-risk surgeries			3 (p=0.060)

## Discussion

Measurement of endothelial function in patients has emerged as a leading option for studying atherosclerosis. Endothelial cells alter the various inflammatory, cellular proliferation, and thrombotic pathways, altering the long-term function of the vasculature [10]. The endothelium loses its normal function of vascular regulation when it is exposed to cardiovascular risk factors. This contributes to clinical syndromes such as acute MI, angina, stroke, or peripheral vascular disease [11]. The severity of endothelial dysfunction has been found to correlate to the increased risk of a cardiac event, according to most studies [12].

Endothelial function is usually measured using its vasodilatory response to a mechanical or a chemical stimulus. Impaired FMD in the brachial and radial artery has been found to correlate with long-term atherosclerosis and cardiovascular events [13]. Endothelial dysfunction has also been shown to precede and further potentiate atherosclerosis [14]. Studies also show that endothelial dysfunction detected by FMD in the brachial artery positively correlated with coronary artery dysfunction [11]. FMD is non-invasive, cheap, and easy to perform and thus very useful in studying both the benefits and harm of any cardiovascular intervention [6].

Atherosclerosis is an intimal disorder of the vessels. Ultrasonography cannot delineate between the intimal and medial layers of the vessel, which is the reason why our measurements included both of the layers [15]. However, several studies have proved that an increased carotid artery intima-media thickness is not only a marker of atherosclerosis in the arterial system elsewhere but also a harbinger of cardiovascular and cerebrovascular events [16,17]. Several cardiovascular risk factors have been associated with an impaired CIMT and increased cardiovascular event risk [18].

The clinical and therapeutic relevance of CIMT is right now limited to the assessment of future cardiac event risk. But studies and trials on the efficacy of certain drugs and interventions using CIMT as an outcome measure may provide results that would have a major say in clinical practice [18].

Our study was conducted with an aim to assess the prevalence of endothelial dysfunction and early atherosclerosis in a cohort of Indian patients with clinical and behavioral risk factors (smoking) of endothelial dysfunction (American Society of Anesthesiologists (ASA) class 2 and 3) and also to identify the association that pre-operative vascular function assessment had on the incidence of perioperative cardiovascular events.

It was found that there was no significant difference in the occurrence of events in patients with impaired and normal FMD. There have been no similar studies that correlated perioperative cardiac events with FMD. However, several studies, including those by Chan et al. [19] and Shechter et al., [20], showed that impaired FMD predicted significantly cardiac events in the population under study over a longer follow-up period. Studies, such as the one by Perticone et al. in hypertensive patients [21], by Neunteufl et al. in coronary artery disease (CAD) patients [22], and Bugiardini et al. in young women with angina and normal coronaries [23], all showed that an impaired FMD significantly predicts cardiac events in the future. However, all these studies had a long follow-up period, unlike our study, which concentrated on the immediate perioperative period till the patient’s discharge from the hospital.

There was no significant increase in the occurrence of perioperative cardiac events in patients with an impaired CIMT. This is in line with the study done by Chan et al., which showed that CIMT didn’t predict any significant increase in long-term cardiovascular events [19]. Bots et al. [18], on the other hand, showed that common carotid IMT significantly predicted cardiovascular events in the future. The same reason for an insignificant FMD finding can be applied here as well, owing to the acute nature of the follow-up period. 

Salonen et al. studied a random sample of middle-aged Finnish men [24] and found that a minute increase in CIMT increased the chances of myocardial infarction (MI) significantly. The reason for the negative results on this front in our study could be explained by the fact that our study had a wide age-group range of 18-65 years, as a result of which the inter-group variation was higher. Moreover, even in the elderly age group, patients were on several medications for their underlying diseases, including statins, which were associated with improvements in both the FMD and CIMT. [11]

High-risk surgeries [25] failed to produce a significant increase in perioperative cardiac events. Huang et al., in their study, assessed the endothelial function of 267 patients with peripheral arterial disease posted for vascular surgery using FMD and reactive hyperemia [13]. The median follow-up was almost a year, and they found that both FMD and reactive hyperemia significantly correlated with adverse cardiac events in the future. The absence of a positive result in our study may be due to the fact that most patients who were taken up for such high-risk surgeries had well-controlled risk factors, failing which they were denied fitness for the surgery. Moreover, the surgical procedures were done by experienced surgeons under the careful monitoring of experienced anesthesiologists leading to minimal surgical and anesthetic complications. The follow-up period was also limited. Moreover, patients undergoing vascular surgeries were excluded from our study.

There was also no significant correlation between FMD and CIMT in our study. This was similar to a study done by Chan et al. [16], where it was found that the FMD and CIMT did not have any significant correlation with each other. However, in a study by Oz et al. [3], it was found that both the parameters significantly correlated with each other. In a study by Kobayashi et al. [26], it was found that CIMT and brachial FMD correlated significantly with each other. They also found that both the parameters taken in combination had a higher predictive value for atherosclerosis. This might possibly be due to the fact that the above-mentioned studies were done in a group of subjects who were all known cases of CAD or established atherosclerosis, while our study was done in a heterogeneous group of patients.

In a study by Juonala et al. on more than 2000 young healthy adults [27], it was found that FMD and CIMT significantly correlated with each other adjusted to other behavioral and clinical risk factors. This could be attributed to the huge sample size of this study, while our study was a pilot study with a much smaller sample size.

The median FMD was not significantly different between patients who had a specific risk factor such as diabetes mellitus (DM), hypertension (HTN), etc., and those who did not. The median CIMT values were also similar between patients having a specific risk factor and those who did not have it. This is in contrast to the study by Dosi et al. which had a significant correlation between impaired FMD and dyslipidemia [28]. One possible reason for this difference is the wide range of clinical risk factors each patient in our study had compared to the above study. Our study had a heterogeneous sample size. Patients were at different levels of their disease and had a combination of multiple clinical risk factors. Patients who were cleared fit for elective surgery had an optimally controlled disease process and hence the FMD may have been normal in such patients, despite the underlying risk factor.

Furthermore, the odds ratio analysis did not show any significant association between the given risk factors and the occurrence of perioperative cardiovascular events. The number of events that were observed in these patients was minimal, leading to poor risk factor analysis.

Limitations of the study

It must be noted that this study had a smaller sample size and a very short period of follow-up due to time constraints. In line with most of the studies mentioned above, if these patients are followed up over a longer period of time, more cardiovascular events would be expected from the same study.

## Conclusions

The prevalence of endothelial dysfunction in our tertiary center was found to be 23%, and the prevalence of atherosclerosis was 33% in patients posted for elective non-cardiac surgery who had multiple cardiovascular risk factors. We also conclude that non-invasive preoperative vascular assessment was not quite effective as hypothesized in predicting perioperative cardiovascular events.

FMD and CIMT evaluate different aspects of the vascular function but of the same arterial tree, and thus, their combination would provide greater accuracy. This was a pilot study with a small group of 100 patients due to time constraints, and this study can very well serve to be the stepping stone for a bigger study over a longer period of time.

## References

[1] Kumari B, Kumar B, Gupta D, Ganju N (2021). FMD and CIMT: surrogate markers of atherosclerosis in subclinical and overt hypothyroidism in sub Himalyan region. Indian J Endocrinol Metab.

[2] Fan CY, Zhang ZY, Mei YF, Wu CJ, Shen BZ (2012). Impaired brachial artery flow-mediated dilation and increased carotid intima-media thickness in rheumatoid arthritis patients. Chin Med J.

[3] Oz F, Elitok A, Bilge AK, Mercanoglu F, Oflaz H (2012). Relationship between brachial artery flow-mediated dilation, carotid artery intima-media thickness and coronary flow reserve in patients with coronary artery disease. Cardiol Res.

[4] Davignon J, Ganz P (2004). Role of endothelial dysfunction in atherosclerosis. Circulation.

[5] Münzel T, Sinning C, Post F, Warnholtz A, Schulz E (2008). Pathophysiology, diagnosis and prognostic implications of endothelial dysfunction. Ann Med.

[6] Charakida M, Masi S, Lüscher TF, Kastelein JJ, Deanfield JE (2010). Assessment of atherosclerosis: the role of flow-mediated dilatation. Eur Heart J.

[7] Jadhav UM, Sivaramakrishnan A, Kadam NN (2003). Noninvasive assessment of endothelial dysfunction by brachial artery flow-mediated dilatation in prediction of coronary artery disease in Indian subjects. Indian Heart J.

[8] Stein JH, Korcarz CE, Hurst RT (2008). Use of carotid ultrasound to identify subclinical vascular disease and evaluate cardiovascular disease risk: a consensus statement from the American Society of Echocardiography Carotid Intima-Media Thickness Task Force. Endorsed by the Society for Vascular Medicine. J Am Soc Echocardiogr.

[9] Jørgensen ME, Torp-Pedersen C, Gislason GH (2014). Time elapsed after ischemic stroke and risk of adverse cardiovascular events and mortality following elective noncardiac surgery. JAMA.

[10] Halcox JP, Donald AE, Ellins E (2009). Endothelial function predicts progression of carotid intima-media thickness. Circulation.

[11] Widlansky ME, Gokce N, Keaney JF (2003). The clinical implications of endothelial dysfunction. J Am Coll Cardiol.

[12] Suwaidi JA, Hamasaki S, Higano ST, Nishimura RA, Holmes DR Jr, Lerman A (2000). Long-term follow-up of patients with mild coronary artery disease and endothelial dysfunction. Circulation.

[13] Huang PH, Chen JW, Lu TM, Yu-An Ding P, Lin SJ (2007). Combined use of endothelial function assessed by brachial ultrasound and high-sensitive C-reactive protein in predicting cardiovascular events. Clin Cardiol.

[14] Halcox JP, Schenke WH, Zalos G (2002). Prognostic value of coronary vascular endothelial dysfunction. Circulation.

[15] Stary HC, Blankenhorn DH, Chandler AB (1992). A definition of the intima of human arteries and of its atherosclerosis-prone regions. A report from the Committee on Vascular Lesions of the Council on Arteriosclerosis, American Heart Association. Arterioscler Thromb.

[16] Bots ML, Witteman JC, Grobbee DE (1993). Carotid intima-media wall thickness in elderly women with and without atherosclerosis of the abdominal aorta. Atherosclerosis.

[17] Polak JF, O'Leary DH, Kronmal RA (1993). Sonographic evaluation of carotid artery atherosclerosis in the elderly: relationship of disease severity to stroke and transient ischemic attack. Radiology.

[18] Bots ML, Hoes AW, Koudstaal PJ, Hofman A, Grobbee DE (1997). Common carotid intima-media thickness and risk of stroke and myocardial infarction: the Rotterdam Study. Circulation.

[19] Chan SY, Mancini GBJ, Kuramoto L, Schulzer M, Frohlich J, Ignaszewski A (2003). The prognostic importance of endothelial dysfunction and carotid atheromaburden in patients with coronary artery disease. J Am Coll Cardiol.

[20] Shechter M, Shechter A, Koren-Morag N, Feinberg MS, Hiersch L (2014). Usefulness of brachial artery flow-mediated dilation to predict long-term cardiovascular events in subjects without heart disease. Am J Cardiol.

[21] Perticone F, Ceravolo R, Pujia A (2001). Prognostic significance of endothelial dysfunction in hypertensive patients. Circulation.

[22] Neunteufl T, Heher S, Katzenschlager R (2000). Late prognostic value of flow-mediated dilation in the brachial artery of patients with chest pain. Am J Cardiol.

[23] Bugiardini R, Manfrini O, Pizzi C, Fontana F, Morgagni G (2004). Endothelial function predicts future development of coronary artery disease: a study of women with chest pain and normal coronary angiograms. Circulation.

[24] Salonen JT, Salonen R (1993). Ultrasound B-mode imaging in observational studies of atherosclerotic progression. Circulation.

[25] Lee TH, Marcantonio ER, Mangione CM (1999). Derivation and prospective validation of a simple index for prediction of cardiac risk of major noncardiac surgery. Circulation.

[26] Kobayashi K, Akishita M, Yu W, Hashimoto M, Ohni M, Toba K (2004). Interrelationship between non-invasive measurements of atherosclerosis: flow-mediated dilation of brachial artery, carotid intima-media thickness and pulse wave velocity. Atherosclerosis.

[27] Juonala M, Viikari JS, Laitinen T, Marniemi J, Helenius H, Rönnemaa T, Raitakari OT (2004). Interrelations between brachial endothelial function and carotid intima-media thickness in young adults: the cardiovascular risk in young Finns study. Circulation.

[28] Dosi RV, Acharya DS, Patell RD (2012). Endothelial dysfunction in a cohort of Indian patients with type-2 diabetes mellitus. JIACM.

